# Ultra-simplified diffraction-based computational spectrometer

**DOI:** 10.1038/s41377-023-01355-4

**Published:** 2024-01-05

**Authors:** Chuangchuang Chen, Honggang Gu, Shiyuan Liu

**Affiliations:** 1 State Key Laboratory of Intelligent Manufacturing Equipment and Technology, Huazhong University of Science and Technology, Wuhan, Hubei 430074 China; 2 Optics Valley Laboratory, Wuhan, Hubei 430074 China

**Keywords:** Near-infrared spectroscopy, Optical metrology

## Abstract

Miniaturizing spectrometers for compact and cost-effective mobile platforms is a major challenge in current spectroscopy research, where conventional spectrometers are impractical due to their bulky footprint. Existing miniaturized designs primarily rely on precalibrated response functions of nanophotonic structures to encode spectral information captured in a snapshot by detector arrays. Accurate spectrum reconstruction is achieved through computational techniques, but this requires precise component design, high-precision fabrication, and calibration. We propose an ultra-simplified computational spectrometer that employs a one-to-broadband diffraction decomposition strategy facilitated by a numerical regularized transform that depends only on the spectrum of the diffracted radiation. The key feature of our design is the use of a simple, arbitrarily shaped pinhole as the partial disperser, eliminating the need for complex encoding designs and full spectrum calibration. Our spectrometer achieves a reconstructed spectral peak location accuracy of better than 1 nm over a 200 nm bandwidth and excellent resolution for peaks separated by 3 nm in a bimodal spectrum, all within a compact footprint of under half an inch. Notably, our approach also reveals a breakthrough in broadband coherent diffractive imaging without requiring any *prior* knowledge of the broadband illumination spectrum, assumptions of non-dispersive specimens, or correction for detector quantum efficiency.

## Abstract (web summary)

Ultra-simplified compact spectrometer with a simple, arbitrarily shaped pinhole as the diffracted disperser, eliminating need for encoding and full spectrum calibration, and achieving better than 3 nm spectral peak resolution.

## Graphical abstract

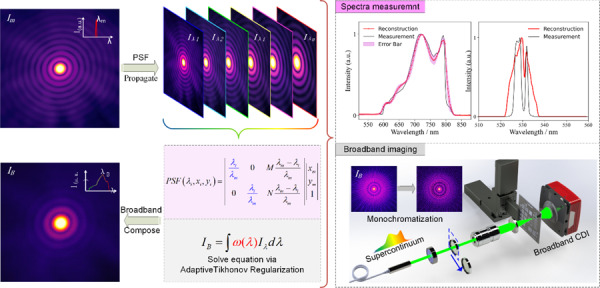

## Introduction

Miniaturized spectrometers, characterized by their compact size and improved performance compared to conventional spectrometers, offer significant potential in various applications such as spectral characterization^1,2^, materials analysis^3^, and hyperspectral imaging^4^. Recent advancements in high-precision lithographic micro-fabrication^5^ and computational techniques^6,7^ have led to the development of a range of miniaturized spectrometers based on nanophotonic dispersive structures or spectral filter sensors. These spectrometers can be broadly categorized into two design approaches. The first approach involves one-to-one spectral-to-spatial mapping, where different bands of the light spectrum are separated spatially or temporally and then detected sequentially by a sensor array. Such spectrometers are generally based on conventional grating-based dispersions^8–11^, meta-surface dispersions^12–15^, waveguide propagations^16–18^, digital planar holography^19,20^, dispersive photonic crystals^21,22^, narrowband filters^23–27^, microfiber taper^28^, and microcrystal resonators^29–32^. However, these instruments typically have limitations in terms of narrowband spectral dispersions across a wide spectrum range and suffer from low photon throughput. An alternative approach relies on broadband-to-broadband spectra mapping combined with computational retrieval algorithms. In this design, the intensities of multiple spectral bands are simultaneously detected after passing through different broadband filters, and the input spectrum is computationally reconstructed. Quantum dot arrays^33,34^, nanowire sensors^35^, and disordered multi-scatterings^36–38^ have been explored in such designs. Nonetheless, the aforementioned systems require meticulous designs, and their performance is highly susceptible to fabrication errors and environmental disturbances. Complex calibration procedures and long-term instability also limit the resolution and robustness of these methods. The representative state-of-the-art computational spectrometers over the past decade are summarized in Table 1.

**Table 1:** Comparison of State-of-the-art Compact Computational Spectrometers

Method	Bandwidth (Δλ/λ_c_)	Spectral resolution	Dispersion	Calibration	Architecture complexity
Nat. Photonics (2013)^37^	1%	0.75 nm	Photonic-crystal	Complex	Complex
Nature (2015)^33^	25%	3 nm	Quantum dots	Ultra-complex	Ultra-complex
Optica (2016)^36^	0.1%	0.01 nm	Multimode spiral	Complex	Ultra-complex
Science (2018)^14^	12%	NA	Metalens	Complex	Ultra-complex
Science (2019)^35^	10%	15 nm	Nanowire	Complex	Moderate
Nat. Photonics (2020)^53^	3.2%	10 nm	Nano-film	Moderate	Complex
Light Sci. Appl.(2021)^54^	35%	5.2 nm	Spectral camera	Ultra-complex	Ultra-complex
ACS Photonics (2022)^55^	3.4%	30 nm	Metalens	Complex	Complex
Light Sci. Appl.(2023)^15^	15%	22 nm	Metalens	Complex	Complex
eLight (2023)^28^	10%	6.89 pm	Microfiber taper	Ultra-complex	Simplified
This Work	28%	3 nm	Pinhole	Ultra-simplified	Ultra-simplified

^*^Δ*λ* = full width of the spectrum at half maximum, *λ*_c_ = center wavelength of the broadband spectrum

Recently, broadband diffraction with partial coherence brought new insight into both coherent diffractive imaging (CDI)^39,40^ and spectrum metrology^41,42^. This principle relies on the linear superposition of a broadband diffraction pattern, composed of coherent diffraction components that are inherently wavelength-dependent and characterized through the propagation of the spatial-spectral point-spread function (PSF) in the source spectrum. However, for diffraction-based spectrometers, the design of diffractive optics for encoding and the characterization of the PSF over the entire spectrum range requires careful calculations. These considerations introduce trade-offs that limit the performance of spectrometers in terms of simplicity and miniaturization.

Here, we report a novel and straightforward spectrometer design based on one-to-broadband diffraction mapping. Our innovative approach incorporates an arbitrarily shaped pinhole as a diffraction-based partial-disperser positioned in front of the detector. This eliminates the need for pre-encoding designs, making the spectrometer ultra-simplified. By solving a multi-variable linear equation (MLE), we can determine the incident light’s spectrum accurately. The MLE is solved using coherent mode decomposition, employing a numerical regularized transform based on a single-shot measurement of quasi-monochromatic diffraction, which serves as the point-spread function (PSF). Importantly, the PSF relies solely on the diffracted radiation spectrum.

Experimental verification of our developed spectrometer demonstrates a reconstructed spectral peak location accuracy better than 1 nm over a 200 nm bandwidth and spectral resolution for a bimodal spectrum with peaks of 3 nm separation, all within a compact footprint of under half an inch. This represents the first demonstration of a spectrometer design that integrates an ultra-simplified and arbitrarily shaped diffraction structure. Our design eliminates the need for pre-encoding designs, high-precision fabrication, or complex calibration processes. It enables single-shot spectrum measurements across a wide wavelength range, from ultraviolet to infrared, with miniaturized lab-on-chip integration. This advancement is crucial for portable applications, offering high robustness, low cost, and long-term stability. Furthermore, the proposed method also reveals a significant breakthrough in broadband CDI without requiring any *prior* knowledge of the broadband illumination spectrum, assumptions of non-dispersive specimens, or correction of detector quantum efficiency (QE).

## Results

### Schematic of diffraction-based spectrometer

**Fig. 1:**
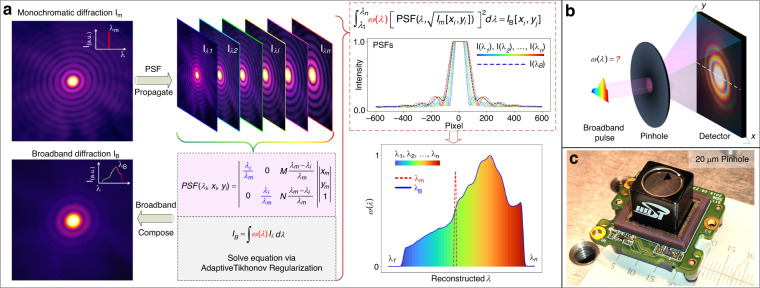
The proposed one-to-broadband diffraction-based spectrometer. **a** Schematic of the operation principle from a monochromatic diffraction to a broadband diffraction, i.e., one-to-broadband diffraction. A broadband diffraction ${I}_{B}$ captured in-situ can be represented as a superposition of wavelength-dependent PSFs from a single-shot monochromatic diffraction ${I}_{m}$ at wavelength ${\lambda }_{m}$ times the corresponding power spectrum components ${\rm{\omega }}\left(\lambda \right)$ over full spectrum. The incident spectrum ${\rm{\omega }}\left(\lambda \right)$ can be reconstructed using adaptive Tikhonov regularization. **b** Geometry of the proposed diffraction-based spectrometer. **c** A photograph of the ultra-compact computational spectrometer developed based on the proposed principle, which is integrated into a tiny CMOS panel (10 mm × 10 mm in size)

A diffraction pattern of a hollow microstructure (with a constant transmission over the whole concerned spectrum) with broadband radiation in an arbitrary state of coherence can be interpreted as a linear superposition of a discrete set of monochromatic diffraction patterns within the source spectrum. The diffraction intensity in each coherent mode is inherently wavelength-dependent and depends on the spectrum of the diffracted radiation^40^. It is possible to retrieve the spectrum from the broadband diffraction pattern with the knowledge of all spectral components of monochromatic diffractions, which can be treated as an ill-posed MLE. This means that the radiation spectrum could be calculated only if the transmittance coefficients of each monochromatic diffraction for different wavelengths are pre-characterized as the encoding information. However, this calibration process is usually cumbersome, sometimes even not achievable in practical applications. The key point of our method is that each individual-wavelength diffraction profile *I*_*λ*_ at wavelength *λ* can be achieved from a single shot of a monochromatic diffraction pattern *I*_*m*_ at a given wavelength *λ*_*m*_ by utilizing the one-to-broadband PSF mapping scheme (Fig. 1a). Applied with this information, the input spectrum can be reconstructed from a single-shot broadband diffraction combined with its corresponding PSFs. For a given application in diffraction optics (Fig. 1b), a wave propagated from an object couples the amplitude and phase of a diffraction field *U*(*x’, y’, 0*) through the Fraunhofer diffraction. Since the detector only records the amplitude of diffraction and drops the phase information, ${I}_{\lambda }$ can be described as^43^:

1 $${I}_{\lambda }(x,y,z)={(\frac{c}{\lambda z}{| {\mathcal F} \{U(x^{\prime} ,y^{\prime} ,0)\}|}_{u=\frac{x}{\lambda z},v=\frac{y}{\lambda z}})}^{2}$$

where propagation travels a distance of *z*, λ is the wavelength of the radiation and ${\mathscr{F}}$ denotes the 2D spatial Fourier transform of the sample *U*(*x’, y’, 0*) at *z* = 0, with *u* and *v* the spatial frequencies. Equation (1) indicates that the Fraunhofer diffraction intensity distribution depends only on the propagation distance *z* and wavelength λ in an identical way, showing a wavelength-dependent scaling factor *c*/*λ*z, which allows us to map a coherent diffraction *I*_*λ*_ at an arbitrary wavelength from a single shot of monochromatic diffraction *I*_*m*_ at a given wavelength *λ*_*m*_ by PSF propagation between different spectral components (Fig. 1a). Owing to the microfeature size of the pinhole, the incident radiation can be considered as a spatial coherent illumination, which preserves a uniformity of the spectral intensity. Since the detector array captures a diffraction pattern with a much longer integration time than the coherence time of radiation, the broadband diffraction pattern *I*_*B*_ can be treated as the incoherent sum of all spectrum components, written as:

2 $${I}_{B}=\int \omega (\lambda ){[PSF(\lambda )]}^{2}d\lambda$$

where, *PSF*(*λ*) represents the sum of PSFs from the reference diffraction *I*_*m*_, and *ω*(*λ*) is the power spectrum of the diffracted light. Equation (2) indicates that a broadband diffraction pattern *I*_*B*_ captured by a detector with *M* *×* *N* pixels can be represented as the integral of *ω*(*λ*)[*PSF*(*λ*)]^2^ over the wavelength range, including of *M* *×* *N* multi-linear simultaneous equations with *n* variables of discrete spectral components over full spectrum range (details in Supplementary S1). Due to the measurement noise in both *I*_*B*_ and *I*_*m*_ combined with the approximation errors in PSF mapping that make the equations ill-posed, it is generally impossible to solve these equations straightforwardly by ordinary noniterative methods. Herein, a least-square-based multi-variable linear regression (MLR) scheme applying an adaptive Tikhonov regularization is employed to reconstruct the power spectrum *ω*(*λ*) and suppress the noises and errors during reconstruction. Additionally, generalized cross-validation (GCV) statistics are applied to balance the requirements of robustness and resolution^44^ (details in Supplementary S2).

Noting that the proposed one-to-broadband diffraction-based computational spectrometer is ultra-simplified and only relies on a single-shot of broadband diffraction *I*_*B*_ of an arbitrary shaped microstructure as input combined with a shot of monochromatic diffraction *I*_*m*_ pre-captured in situ without any movement parts or complex calibrations over full spectrum, it is designed with an ultra-compact form. As shown in Fig. 1c, the experimental prototype of such spectrometer was formed by coupling a Φ20 μm pinhole in front of the CMOS array detector. The system was integrated into a digital camera, with a size comparable footprint of 10 mm × 10 mm in size.

### Experimental validation

To illustrate the performance of the proposed spectrometer, a group of experimental validations was performed using a supercontinuum light source (YSL Photonics SC-Pro-M) and a set of optical filters (Thorlabs F series) to modulate the shape of incident spectra. The light scattered from a Φ20 μm pinhole was recorded by a digital camera (MV-CA050) to capture a coherent quasi-monochromatic diffraction shot *I*_*m*_ from a narrowband spectrum and a broadband diffraction shot *I*_*B*_ in situ from an unknown wide spectrum, respectively (set-up detailed in Supplementary S3).

**Fig. 2:**
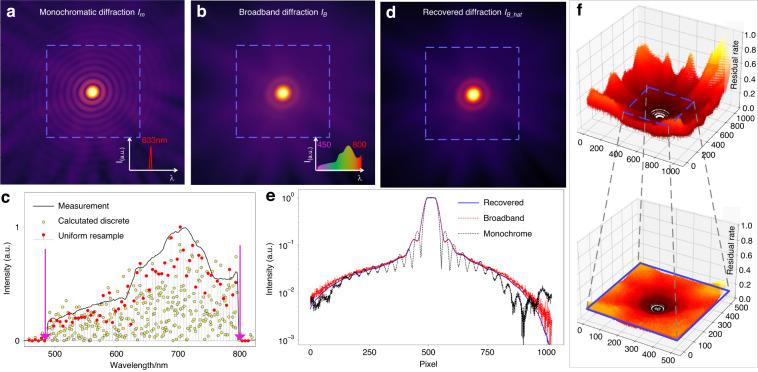
Superposition of broadband diffraction via PSF mapping. **a** The coherent monochromatic diffraction *I*_*m*_ by a 633 nm band-filter. **b** The broadband diffraction *I*_*B*_ captured by a wide spectrum illumination. **c** The measured spectrum (solid curve), the calculated power spectrum components (yellow dots), and the linear fitting with a uniform interval of 5 nm (red dots). **d** Recovered diffraction *I*_*B_hat*_ from the calculated spectra power scatters. **e** Vertical line cuts along the center of the diffraction patterns, and red, black, and blue curves correspond to the patterns in subfigures **a**, **b**, and **ds**, respectively. **f** 3D distribution of an RR map between the measured diffraction *I*_*B*_ and its prediction *I*_*B_hat*_, and the insert shows the zoom-in RR map in the region of blue boxes. All diffraction patterns are scaled to 1/2 power

To verify the superposition of broadband diffraction via PSF mapping, we first captured a coherent monochromatic diffraction shot *I*_*m*_ (Fig. 2a) in situ at a 633 nm wavelength to generate a series of PSFs as the regressors over full band spectrum components and a shot of broadband diffraction *I*_*B*_ (Fig. 2b) from radiation with 350 nm bandwidth spectrum (Fig. 2c solid curve) as the spectrum-response variable. Then, 334 discrete counts of the power spectrum *ω*(*λ*) were calculated at different wavelength positions by solving Eq. (2) with the MLR scheme (Fig. 2c yellow dots). Then, a linear fitting with a uniform interval of 5 nm is processed to resample the calculated dataset (Fig. 2c red dots). Note that the distribution of the calculated power spectrum presents a high alignment with the measurement, even notably when subjected to specified step changes of the power spectrum. Thereafter, we recovered a prediction of the broadband diffraction *I*_*B_hat*_ by summing up the calculated spectral components times the corresponding PSFs (Fig. 2d). The recovered diffraction pattern matches well with the measured broadband diffraction, especially in the low orders of diffraction, while the intensities in high orders are blurred due to the inherent nature of temporal decoherence and the read noise of camera (Fig. 2e). Moreover, we used the residual rate (RR) (*I*_*B*_ − *I*_*B_hat*_)/*I*_*B*_ to evaluate the alignment between the captured broadband diffraction and its prediction (Fig. 2f). Seeing that the RR increases along with the order of the diffraction since only first several orders of broadband diffraction are recorded with high SNR (signal-to-noise ratio), corresponding to RR < 0.1.

**Fig. 3:**
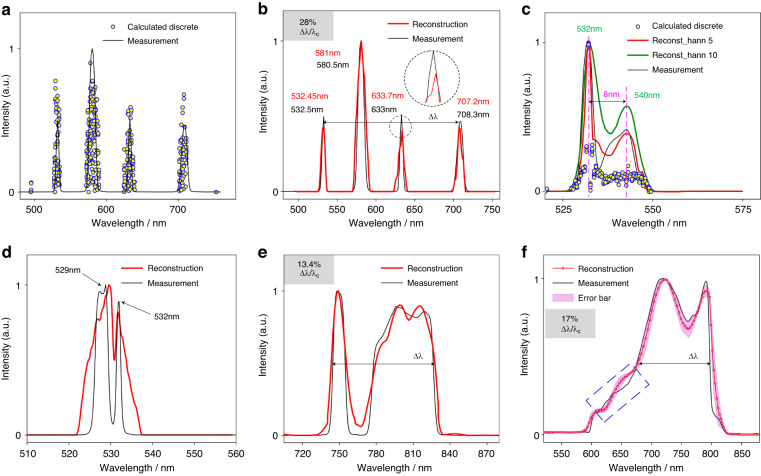
Comparison between the reconstructed spectra (red curves) and the measurements (black curves) from a commercial grating-based spectrometer (Horiba iHR550). It should be noticed that all these results are normalized to their peak intensities and the QE coefficient of the detector is eliminated from the reconstructions. **a** Calculated discrete scatters (yellow circles) of a spectrum with 4 narrowband peaks corresponding to 532 nm, 580 nm, 633 nm, and 710 nm, compared with the measurement. **b** The reconstructed spectrum (red line) from the calculated discrete data in (**a**). **c** Calculated discrete scatters and the reconstructions of a bimodal spectrum with peaks of 8 nm apart under different Hann kernel sizes compared with the measurement. **d** The reconstruction of a bimodal spectrum with peaks of 3 nm separation. **e**, **f** Reconstructions of two different broadband spectra modulated by filters from a supercontinuum light source with the bandwidth of 100 nm and 230 nm, respectively. In **f**, the mean spectrum of five trials of measurements and the corresponding error bar (standard deviation)

Figure 3 presents five arbitrary spectra reconstructed by the above process (red curves) compared with the corresponding measurements (black curves). It should be mentioned that a quasi-monochromatic diffraction *I*_*m*_ from a given narrowband filter is pre-captured to generate the PSFs before each spectrum reconstruction, as mentioned above. Results in Fig. 3a were reconstructed from the corresponding pre-captured pattern *I*_*m*_ at the wavelength of 633 nm with a full width at half maximum (FWHM) of 3 nm, Fig. 3c, d from that at the wavelength of 532 nm with an FWHM of 3 nm and 1 nm, respectively, Fig. 3e from that at the wavelength of 750 nm with a FWHM of 10 nm, and Fig. 3f from that at the wavelength of 710 nm with a FWHM of 10 nm, respectively. In the first case, a wide spectrum with 4 narrowband peaks around the wavelength of 532 nm, 580 nm, 633 nm, and 710 nm was concerned, which was reconstructed from these calculated power spectrum components (Fig. 3a) via uniform resampling and convolution procedures. It indicates that the reconstructed spectrum exhibits a peak location accuracy better than 1 nm over the concerned 200 nm spectral range (Fig. 3b). In practice, the convolution with a Hann window is used to process the reconstructed spectrum, and the Hann kernel shapes the spectrum curves enormously (Fig. 3c), resulting in notable mismatch to the measurement in ground truth. It indicates that the convolution kernel should be carefully selected to improve the precision of spectrum reconstruction in practical applications. Furthermore, as illustrated in Fig. 3d, the proposed spectrometer presents a high spectral resolution and can easily distinguish a bimodal spectrum with peaks of 3 nm separation from the corresponding pre-captured narrowband diffraction with a FWHM of 1 nm. Moreover, to verify the robustness against the bandwidth, reconstructions of arbitrarily shaped broadband spectra with bandwidths of 100 nm and 230 nm were carried out, and results are respectively shown in Fig. 3e, f. Note that the reconstructed spectral peaks agree well with the measurements in ground truth by a commercial grating-based spectrometer (Horiba iHR550), and the spectral curves are also well-traced with relatively low error bars. Tiny mismatches occur occasionally on steep turning points of the spectra when subjected to ultra-broadband spectrum, as indicated with the blue dashed box in Fig. 3f.

### Spectrum reconstruction quality

The proposed one-to-broadband diffraction-based computational spectrometer is based on PSF mapping. Therefore, ideally, the spectral resolution is limited by the spectral sampling interval of the PSF mapping for a constant diffraction distance *z*:

3 $$\delta \lambda =\frac{{{\lambda }_{m}}^{2}}{({\lambda }_{1}+{\lambda }_{n}){n}_{{pixel}}}\approx \frac{{\lambda }_{m}}{2{n}_{{pixel}}}$$

where *n*_*pixel*_ is the sum of pixels within the active sensor array size in the detector, *λ*_1_ and *λ*_*n*_ denote the boundaries of the broadband spectrum. Equation (3) indicates that the spectral resolution is reciprocally related to the number of sampling pixels in the active detector sensor array, primarily determined by the detector’s dynamic range. Since higher diffraction orders typically exhibit lower intensity, resulting in poor SNR. To address this, we intentionally overexpose and subsequently filter out the zero-order diffraction to fully utilize the sensor’s dynamic range. Additionally, we eliminate background noise in the detector to enhance the diffraction SNR. In our diffraction-based spectrometer, utilizing larger active sensor arrays enhances spectrum measurement precision but also comes with a significant increase in computational expense. Consequently, we carefully bin the pixels to strike a balance between the quality of spectrum measurement and computational expenses. For a more detailed analysis, please refer to Supplementary S4.

**Fig. 4:**
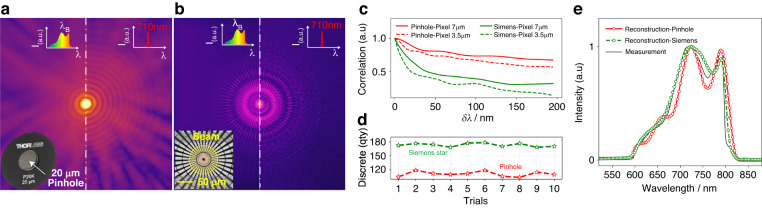
Diffraction efficiency of the diffracted disperser boosts spectral measurement quality. **a**, **b** Broadband (left) and narrowband (right, wavelength of 710 nm with 10 nm FWHM) diffractions of a pinhole and a Siemens star, respectively. **c** The calculated spectral correlation function of the PSF for different diffraction devices and pixel sizes of the detector. **d** The counts of the calculated spectrum components over 10 trails of calculations from the diffraction data of the Siemens star and pinhole, respectively. **e** Reconstructed spectra for the Siemens star and the pinhole, corresponding to a pixel size of 7 μm, compared with the measurement (black curve)

In practical applications, the reconstructed spectrum is usually more sensitive to the diffraction efficiency of the disperser device. We further conducted two groups of validations by applying a Φ20 μm pinhole and a Φ100 μm Siemens star as the diffracted disperser, respectively. Noting that the diffractions from the Siemens star (Fig. 4b) retain more abundant information in frequency with high SNR than these from the pinhole (Fig. 4a), which can improve the generalization ability and the accuracy of the proposed diffraction-based computational spectrometer. here, we introduce the spectral correlation function of the PSF to determine the spectral resolution, as given by:

4 $$C(\delta \lambda )=\frac{{\mathrm{cov}}(PSF({\lambda }_{m}),PSF({\lambda }_{m}+\delta \lambda ))}{\sigma (PSF({\lambda }_{m}))\sigma (PSF({\lambda }_{m}+\delta \lambda ))}$$

Figure 4c plots the spectral correlation functions $C(\delta \lambda )$ of the PSFs for different pixel sizes and diffraction devices as a function of $\delta \lambda$ in wavelength space. Seeing that the spectral correlation functions of the Siemens star drop more steeply than those of the pinhole, the decrease in pixel size will also be helpful in reducing the spectral correlation function. Since the PSFs are performed as the regressors over the full spectral range in the proposed MLR scheme, that’s to say, the lower the correlation of the PSFs, the more spectrum power components can be recovered. Experimental results shown in Fig. 4d, e provide evidence to support this point. Figure 4d comparatively shows the counts of the calculated spectrum components over 10 trails of calculations from the Siemens star and the pinhole, respectively. it can be observed that 180 discrete counts are calculated from the Siemens star, whereas only about 120 counts from the pinhole, which eventually results in a much more precise reconstructed spectrum for the Siemens star than that for the pinhole, as shown in Fig. 4e. The refined design of the diffraction disperser boosts diffraction efficiency, consequently enhancing the quality of spectrum reconstruction. Meanwhile, the spectral response bandwidth of the spectrometer is predominantly limited by the detector QE.

Additionally, the resolution of peak separation of the bimodal spectrum may also degenerate from the decoherence of the pre-captured diffraction, which is used to generate the PSFs over the full spectrum range. The better the coherency of the quasi-monochromatic diffraction, the higher the resolution of spectral peak separation (details in Supplementary S5).

## Discussion

**Fig. 5:**
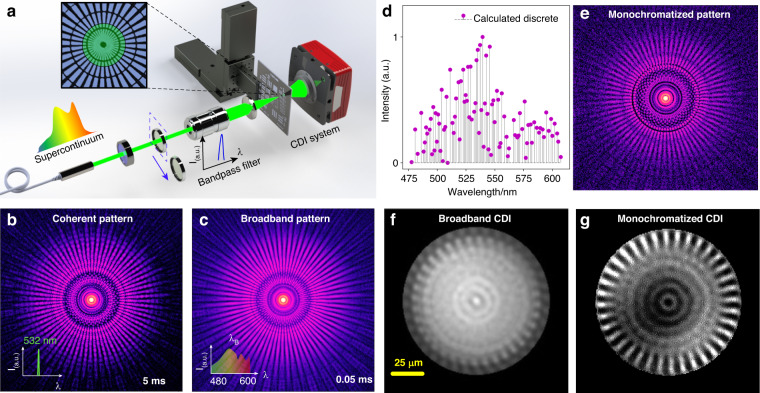
A typical application of broadband CDI using the proposed diffraction-based computational spectrometer. **a** The schematic layout of the broadband CDI set-up. **b** The pre-captured coherent pattern at 532 nm with 5 ms exposure time. **c** The corresponding broadband pattern captured in situ with up to 20% bandwidth (spectrum ranging from 480 nm to 600 nm) with only 0.05 ms exposure time. **d** The aftermost calculated spectrum components of the system at the detector plane from the diffraction datasets given in (**b**, **c**). **e** The monochromatized pattern recovered from the broadband data in (**c**) applying the calculated spectrum components in (**d**). **f** CDI results from the broadband data in (**c**). **g** CDI results from the monochromatized data in (**e**). All diffraction patterns are log-scaled

A fancy application of broadband lensless imaging was further implemented to demonstrate the prospect of the proposed novel spectrometer in practical applications. Figure 5 shows the layout of a broadband CDI set-up (details in Supplementary S6) and the reconstructions by utilizing the developed approach compared with results based on conventional phase retrieval techniques. It should be mentioned that the strict requirement of narrowband radiation with high coherency in current CDI architectures poses a significant obstacle to achieving efficient photon utilization across the full spectrum^45^. Numerous studies have been conducted to overcome this trade-off for broadband CDI in recent years^39,40,46–50^, but encounter several formidable challenges, including the stringent constraints for non-dispersive specimens over full spectrum, the need for accurate spectrum measurement as input, and the requirement for the solutions to converge within the band limit to be valid. These issues severely hamper the advancement in CDI for the ultra-wide spectrum. At the same time, the proposed approach can break through these limitations and enable high-quality CDI even using broadband (i.e., incoherent) illumination without the requirement of any *prior* spectra (e.g., broadband illumination, the spectral transfer function of the specimen, or detector QE).

The aftermost transmission spectrum of the system at the detector plane (Fig. 5d) can be successfully resolved from the pre-captured coherent quasi-monochromatic diffraction pattern (Fig. 5b) and the broadband diffraction pattern in-situ with a bandwidth of 20%, spanning from 480 nm to 600 nm (Fig. 5c) by the proposed method. Then, as demonstrated in Fig. 5e, the monochromatized diffraction pattern can be retrieved from the corresponding broadband diffraction utilizing a numerical monochromatization method^39^ (details in Supplementary S7). Noting that the broadband pattern displayed a significant reduction in coherence compared to the coherent pattern. This decrease in coherence ultimately resulted in a convergence failure during the CDI reconstruction process (Fig. 5f). However, the monochromatization by the proposed method effectively addresses this decoherence issue arising from the broadband radiation. Furthermore, a comparison of CDI results was eventually performed by 500 iterations of RAAR^51^ from the broadband pattern and monochromatized pattern, respectively. Compared with the CDI result from the broadband pattern (Fig. 5f), the monochromatized CDI (Fig. 5g) showcases a remarkable improvement in the quality of the reconstruction with high fidelity. The good spectral agreement and high quality of the broadband CDI results demonstrate the superiority of the proposed approach, implying huge potential applications not only in broadband spectrum metrology with high resolution but also in fields of rising computational imaging techniques.

In summary, we have proposed a novel scheme of computational spectrometer based on the one-to-broadband diffraction applying a simplified and arbitrarily shaped diffraction microstructure as the disperser, which makes the device ultra-compact and low-cost and paves the way towards single-shot spectrum metrology. Different from other computational spectrometer designs, the proposed spectrometer is based on the PSF mapping from a single shot of pre-captured coherent monochromatic diffraction to generate a full spectral response function, and it does not require pre-encoding design, complex fabrication with high precision, or full spectral response function calibration. Experiments conducted on a proof-of-concept have verified the methodology, and results indicate that the proposed computational spectrometer provides a spectral resolution better than 3 nm and the accuracy of the spectral peak is better than 1 nm over a 200 nm bandwidth. Benefiting from its generality of principle, simple architecture, and compact size, the proposed approach has great potential in a huge range of applications in broadband spectrum metrology and computational imaging with miniaturized, cost-effective, and lab-on-chip integration. A broadband CDI prototype is successfully implemented based on the proposed approach to practically demonstrate a fancy application in broadband lensless imaging, which successfully tackles all the challenges of the current state-of-the-art broadband phase retrieval techniques (e.g., the ultra-broadband illumination with unknown spectrum, free of spectral correction of the specimen or detector QE). The proposed method can be easily extended and applied to optical techniques and systems related to multi-state coherent diffraction superposition, such as phase-retrieval-based computational imaging systems with broadband or multi-wavelength illumination.

## Materials and methods

### PSFs calculation

The bandwidth of the incident spectrum is uniformly divided into *n* slices *λ*_1_, *λ*_2_,…, *λ*_*n*_, the PSF at an arbitrary wavelength *λ*_*i*_ distributes as a scale from a monochromatic diffraction pattern *I*_*m*_ at a wavelength *λ*_*m*_:

5 $$PSF({\lambda }_{i},{x}_{i},{y}_{i})=\frac{c}{{\lambda }_{i}z}\left[\begin{array}{ccc}{\lambda }_{i}/{\lambda }_{m} & 0 & M({\lambda }_{m}-{\lambda }_{i})/{\lambda }_{m}\\ 0 & {\lambda }_{i}/{\lambda }_{m} & N({\lambda }_{m}-{\lambda }_{i})/{\lambda }_{m}\end{array}\right]\left[\begin{array}{c}{x}_{m}\\ {y}_{m}\\ 1\end{array}\right]$$

where *x*_*i*_, *y*_*i*_ denotes the coordinates of the diffraction pattern at a wavelength *λ*_*i*_, and *M*, *N* is the total number of pixels in the captured diffraction pattern along the *X*, *Y* direction, respectively. Seeing that the *PSF*(*λ*_*i*_) is an affine transformation from a reference diffraction field $\sqrt{{I}_{m}}$ where *λ*_*i*_*/λ*_*m*_ is the scaling factor to describe the PSF mapping and (*M*(*λ*_*m*_ *−* *λ*_*i*_)/*λ*_*m*_, *N*(*λ*_*m*_ *−* *λ*_*i*_)/*λ*_*m*_) is the translation factor to center the scaled diffraction orders. It is worth noting that since each pixel’s readout value represents the sum of diffraction intensities across all spectral channels, the PSF mapping matrix should be resampled by performing interpolation such that the spatial resolution of *PSF*(*λ*_*i*_) matches the sensor pixel size. Additionally, *λ*_*m*_ would be carefully selected around the mass of the center of the broadband spectrum to reduce the PSF mapping error from interpolation (details in Supplementary S8).

### Spectrum reconstruction

A broadband diffraction *I*_*B*_ is the integral of *ω*(*λ*)[*PSF*(*λ*_*i*_)]^2^ over the wavelength range, which can be performed as an MLR model by summing *n* discrete slices in spectra range for approximation, rewritten to a matrix form in simplicity:

6 $${I}_{B}=\mathop{\sum }\limits_{{\rm{i}}=1}^{n}\omega ({\lambda }_{i}){[PSF({\lambda }_{i})]}^{2}\mathop{\Rightarrow }\limits^{\rm{Simplicity}}{\boldsymbol{A}}{\boldsymbol{\omega }}={\boldsymbol{b}}$$

Note that the formula of Eq. (6) is a system of *M* *×* *N* multi-linear simultaneous equations with $n$-dimensional parameter vector, where ***A*** is a given *M* *×* *N* *×* *n* matrix with elements of each column of a flattened *PSF*(*λ*_*i*_) matrix in 1D array corresponding to the *i*th slice of spectrum and ***b*** is a known vector of a recorded broadband diffraction flattened in 1D array, ***ω*** is the vector of unknown spectrum coefficients for the function. Practically, it is usually impossible to solve the MLR by ordinary noniterative methods due to its ill-posed nature. To tackle such instabilities, a method of residual norm minimization is applied with a weighting regularization factor, as known as Tikhonov regularization, to reconstruct the power spectrum ***ω*** and suppress the noise signals during reconstruction. The least square of the sum of squared residuals with a regularization item is minimized as

7 $$\hat{{\boldsymbol{\omega }}}=\mathop{argmin}\limits_{\omega }{\Vert {\boldsymbol{A}}{\boldsymbol{\omega }}-{\boldsymbol{b}}\Vert }_{2}^{2}+{\varGamma }^{2}{\Vert {\boldsymbol{\omega }}\Vert }_{2}^{2},\,\varGamma \,>\, 0$$

where Г is the regularization coefficient, ‖.‖_2_ is the *l*_2_ norm. Note that the efficiency of these estimates depends on the appropriate choice of the regularization coefficient Г, which should be carefully selected to balance the results of robustness and resolution. Here, we use the GCV statistic to select the regularization coefficient adaptively^52^. As a result, we can have the power spectrum estimates $\hat{{\boldsymbol{\omega }}}$ from Tikhonov regularization Eq. (7).

Since the total components in a measurement of power spectrum $\hat{{\boldsymbol{\omega }}}$ distributes sparsely, corresponding to its spectral sampling interval $\delta \lambda$ in Eq. (3), a linear fitting with a uniform interval is processed to resample the calculated dataset. Finally, the finer reconstruction of an incident spectrum is optimized by a convolution operator with a Hann window to suppress high-frequency interference. The workflow of the proposed computational diffraction-based microspectrometer is detailed in Supplementary S2.

### Supplementary information

SUPPLEMENTAL MATERIAL

## Data Availability

The data and codes that support the plots within this paper and other findings of this study are available from the corresponding author upon reasonable request. Source data are provided in this paper.

## Code availability

The data and codes that support the plots within this paper and other findings of this study are available from the corresponding author upon reasonable request. Source data are provided in this paper.
