# Supplementary material for: Ultra-simplified diffraction-based computational spectrometer
Source: Light Sci Appl. 2024 Jan 5;13:9. doi: 10.1038/s41377-023-01355-4 (PMC10766968; doi:10.1038/s41377-023-01355-4)
Supplement: Supplementary file 1 — SUPPLEMENTAL MATERIAL [file 41377_2023_1355_MOESM1_ESM.docx]

**Supplementary Information for**

**Ultra-simplified diffraction-based computational spectrometer**

Chuangchuang Chen^a^, Honggang Gu^a,b,*^, Shiyuan Liu^a,b,*^

*^a^ State Key Laboratory of Intelligent Manufacturing Equipment and Technology, Huazhong University of Science and Technology, Wuhan, Hubei 430074, China*

*^b^ Optics Valley Laboratory, Wuhan, Hubei 430074, China*

*^*^ Corresponding authors:* [*hongganggu@hust.edu.cn*](mailto:hongganggu@hust.edu.cn) *(H. Gu);* [*shyliu@hust.edu.cn*](mailto:shyliu@hust.edu.cn) *(S. Liu)*

**S1 Broadband Fraunhofer diffraction approximation from PSF superposition**

Consider a monochromatic plane wave with a wavelength $\lambda$ propagated from a hollow microstructure (constant transmission over full spectrum) couples the amplitude and phase of a diffraction field $\psi_{\lambda}\left( x,y,z \right)$ by traveling a distance of $z$, in the paraxial approximation, given by the Fraunhofer diffraction formula^1^:

 (S1)

where $\mathcal{F}$ denotes the 2D spatial Fourier transform of the exit wave function $U\left( x^{'},y^{'},0 \right)$ at z = 0, with $u$ and $v$ the spatial frequencies. In case of broadband radiation, the broadband diffracted field $\Phi$ can be written as:

$\Phi(\lambda)=\sqrt{\omega\left(\lambda\right)}\psi_{\lambda}(x,y,z)$ (S2)

where $\omega\left( \lambda\right)$ is the power spectrum density of the incident radiation. The broadband diffraction field in spatial domain can be obtained by an inverse Fourier transform:

$\Phi(t)=\frac{1}{\sqrt{2\pi}}\int\Phi(\lambda)e^{i\lambda t}d\lambda$ (S3)

Since only the amplitude of diffraction is recorded by the detector, while the phase information is dropped, the detector integrates over time to produce the broadband diffraction pattern $I_{B}$:

$I_{B}=\int\left|\Phi(t)\right|^{2}dt=\frac{1}{2\pi}\int\left|\Phi(\lambda)\right|^{2}d\lambda$ (S4)

with using Parseval’s theorem. By Eq. (S2) substituted into Eq. (S4), we have $I_{B}$:

$I_{B}=\frac{1}{2\pi}\int\omega\left(\lambda\right)\left|\psi_{\lambda}\right|^{2}d\lambda$ (S5)

Known that a recorded monochromatic diffraction pattern $I_{\lambda}$ can be written as:

 (S6)

Seeing that Fraunhofer diffraction intensity distribution depends only on the propagation distance $z$ and wavelength $\lambda$ in an identical way, showing a wavelength-dependent factor $c/\lambda z$, which allows us to map a coherent diffraction $I_{\lambda}$ at an arbitrary wavelength from a single coherent diffraction shot $I_{m}$ at a given wavelength $\lambda_{m}$ by PSF propagation between different spectral components. Introducing the scaling factor ${\lambda_{i}}/{\lambda_{m}}$, the PSF mapping can be described as:

$PSF\left(\lambda_{i},x_{i},y_{i}\right)=\frac{c}{\lambda_{i}z}\begin{matrix} \frac{\lambda_{i}}{\lambda_{m}} & 0 & M\frac{\lambda_{m}-\lambda_{i}}{\lambda_{m}} \\ 0 & \frac{\lambda_{i}}{\lambda_{m}} & N\frac{\lambda_{m}-\lambda_{i}}{\lambda_{m}} \end{matrix}\left|\begin{matrix} x_{m} \\ y_{m} \\ 1 \end{matrix}\right|$ (S7)

where $x_{i},y_{i}$ denotes the coordinates of the diffraction field $\left| \psi_{i} \right|$ at a wavelength $\lambda_{i}$, and $M, N$ is the total number of pixels in the captured diffraction pattern. Seeing that the $PSF\left( \lambda_{i} \right)$ is an affine transformation from a reference diffraction filed $\sqrt{I_{m}}$ where ${\lambda_{i}}/{\lambda_{m}}$ is the scaling factor to describe the PSF mapping and (${M\left( \lambda_{m}-\lambda_{i} \right)}/{\lambda_{m}}$, ${N\left( \lambda_{m}-\lambda_{i} \right)}/{\lambda_{m}}$) is the translation factor to center the scaled diffraction orders.

Thus, combined with Eq. (S5~S7), the broadband diffraction pattern $I_{B}$ can be approximately rewritten as an integration of PSFs from the reference diffraction filed $\sqrt{I_{m}}$, weighted by the power spectrum $\omega\left( \lambda\right)$ over full spectral bandwidth of radiation:

$I_{B}=\int\omega(\lambda)\left[PSF(\lambda)\right]^{2}d\lambda$. (S8)

**S2 Spectrum measurement workflow from a single-shot broadband diffraction**

**Input:**

- $I_{m}$: Pre-captured quasi-monochromatic diffraction pattern at a wavelength $\lambda_{m}$ (Fig. S1a).
- $\mathrm{QE}\left( \lambda\right)$: Detector’s absolute QE (Fig. S1d).

**Step 1: Single-shot broadband diffraction measurement**

Capture a single-shot broadband diffraction pattern $I_{B}$ at an unknown wide spectrum, as shown in Fig. S1**b**. Noting that the zero-order diffractions are overexposed and the background noise of detector is removed to better utilize the detector sensor's dynamic range^2^, the central fringe saturation is consequently filtered from the detector before data processing, as demonstrated in Fig. S1 **a, b**.

**Figure S1 Inputs for the spectrum measurement. a,** the pre-captured quasi-monochromatic diffraction pattern $I_{m}$ at 532 nm with 3 nm FWHM. **b**, the Measured broadband diffraction from unknown spectrum radiation. **c** plots vertical line cuts along the center of the diffraction patterns in **a** and **b**, respectively. **d**, the absolute QE of the detector with spectral response >0.2 ranging from 400nm to 800nm

**Step 2: Calculate PSFs**

Give an initial prediction of the spectrum range, where the bandwidth is usually large than the ground truth. Then, the spectrum is divided uniformly into *n* slices $\lambda_{1},\lambda_{2},{\ldots\lambda}_{n}$ by the interval $\delta\lambda$ uniformly. Practically, $\delta\lambda$ is limited by the detector pixel size $d_{p}$, the recorded diffraction length $D$ with high SNR, and the referenced wavelength $\lambda_{m}$, given by:

$\delta\lambda=\frac{\lambda_{m}d_{p}}{D}$ (S9)

Then, a series of PSFs is calculated from the pre-captured $I_{m}$ by Eq. (S7). Figure S2 plots the distribution of n slices of calculated PSFs in 1D case over ranges of spectrum components. It is worth noting that the PSF mapping matrix should be resampled by performing interpolation such that the spatial resolution of $PSF\left( \lambda_{i} \right)$ matches the sensor pixel size. In this work, a linear interpolation^3^ is performed to resample the PSFs with the interval steps the same as the pixel size of the detector.

**Figure S2** Distribution of PSFs in 1D-case by scanning the wavelength over ranges of spectrum components.

**Step 3 Solving spectrum via MLR scheme**

Due to the measurement noise in both $I_{B}$ and $I_{m}$ combined with the approximation errors in PSF mapping which make the Eq. (S8) ill-posed, it is generally impossible to solve these equations straightforwardly by ordinary noniterative methods. Note that the formula of Eq. (S8) is a system of $M\times N$ multi-linear simultaneous equations with n-dimensional parameter vector. We rewrite Eq. (S8) to a matrix form in simplicity as:

$A\omega=b$ (S10)

where

$$b=\left[\begin{matrix} I_{B}\left(x_{1},y_{1}\right) & I_{B}\left(x_{1},y_{2}\right) & \cdots& I_{B}\left(x_{1},y_{N}\right) & \cdots\end{matrix}I_{B}\left(x_{M},y_{N}\right)\right]^{T}$$

$$A=\left[\begin{matrix} \left[PSF\left(x_{1},y_{1},\lambda_{1}\right)\right]^{2} & \left[PSF\left(x_{1},y_{1},\lambda_{2}\right)\right]^{2} & \cdots& \left[PSF\left(x_{1},y_{1},\lambda_{n}\right)\right]^{2} \\ \left[PSF\left(x_{1},y_{2},\lambda_{1}\right)\right]^{2} & \left[PSF\left(x_{1},y_{2},\lambda_{2}\right)\right]^{2} & \cdots& \left[PSF\left(x_{1},y_{2},\lambda_{n}\right)\right]^{2} \\ \vdots& \vdots& \ddots& \vdots\\ \left[PSF\left(x_{1},y_{N},\lambda_{1}\right)\right]^{2} & \left[PSF\left(x_{1},y_{N},\lambda_{2}\right)\right]^{2} & \cdots& \left[PSF\left(x_{1},y_{N},\lambda_{n}\right)\right]^{2} \\ \vdots& \vdots& \ddots& \vdots\\ \left[PSF\left(x_{M},y_{N},\lambda_{1}\right)\right]^{2} & \left[PSF\left(x_{M},y_{N},\lambda_{2}\right)\right]^{2} & \cdots& \left[PSF\left(x_{M},y_{N},\lambda_{n}\right)\right]^{2} \end{matrix}\right]$$

$$\omega=\left[\begin{matrix} \omega\left(\lambda_{1}\right) & \omega\left(\lambda_{2}\right) & \cdots& \omega\left(\lambda_{n}\right) \end{matrix}\right]^{T}$$

Note that Eq. (S10) is a form of MLR scheme, which is often fitted by minimizing a penalized version of the least squares cost function, as known as Tikhonov regularization^4^, to reconstruct the power spectrum $\boldsymbol{\omega}$ and suppress the noise signals during reconstruction, given by

$\hat{\omega}=\mathop{\mathrm{argmin}}\limits_{\omega} \left\|A\omega-b\right\|_{2}^{2}+\Gamma^{2}\left\|\omega\right\|_{2}^{2}, \Gamma>0$ (S11)

where $\Gamma$ is the regularization coefficient, $\left\| . \right\|_{2}$ is the $l_{2}$ norm. Since the efficiency of these estimates depends on an appropriate choice of the regularization coefficient $\Gamma$, which should be carefully selected to balance the results of robustness and resolution. In this work, we employ a GCV (Generalized Cross-Validation) statistic to make the balanced choice of $\Gamma$ adaptively^5^:

$\hat{\Gamma}=\mathop{\mathrm{argmin}}\limits_{\Gamma} \frac{\left\|A\hat{\omega}-b\right\|_{2}^{2}}{\left[n-Tr\left(A\left(A^{T}A+\Gamma^{2}I\right)^{-1}A^{T}\right)\right]^{2}}$ (S12)

where $I$ is the identity matrix and the operator $Tr$ sums elements on the main diagonal of a matrix. As a result, we can have the power spectrum estimates $\hat{\boldsymbol{\omega}}$ from solving Eq. (S11)

$\hat{\omega}=\left(A^{T}A\mathbf{+}\Gamma^{T}\Gamma\right)^{-1}A^{T}b$ (S13)

a

b

**Figure S3 a**, the distribution of PSFs in 1D-case by scanning the wavelength over ranges of spectrum components. **b**, the resampled results from **a** with a uniform interval $\Delta\lambda$

Note that the final power spectrum is obtained by distorting the detector’s QE $\omega\left( \lambda\right)=\hat{\boldsymbol{\omega}}\boldsymbol{/} QE\left( \lambda\right)$. Fig. S3**a** plots the calculated discrete power spectrum solutions. Since these scatters distribute irregularly along the wavelength axis, we resample the results with a uniform interval $\Delta\lambda$, which is several times of initial interval $\delta\lambda$ in Eq. (S9), as seen in Fig. S3**b**.

**Step 4 Optimization via convolution process**

We perform a convolution operator to the reconstructed data from step 3 with a Hann window to suppress high-frequency interference and reduce the effects of spectral leakage.

$S\left(\lambda\right)=\hat{\omega}\left(\lambda\right)*h\left(\lambda\right)$ (S14)

where * denotes convolution operator, $h\left( \lambda\right)$ is a Hann kernel with a size of $N$ as:

$h\left(\lambda\right)=\frac{1}{2}\left(1-\cos\left(\frac{2\pi\lambda}{N-1}\right)\right)$ (S15)

a

b

Hann Window

Fourier

Transform

c

d

**Figure S4 a**, optimized result (red) which is convolved from the calculated resampled dataset (blue dots) with a Hann widow. **b,** a comparison of optimized results with different sizes of Hann kernels in the convolution process. The shape of Hann window and the corresponding Fourier transform is plotted in **c** and **d**, respectively.

Fig. S4**a** plots the results from a Hann windowing procedure. Seeing that the power spectrum profiles are accurately reconstructed and match well with the measurement. The high-frequency fluctuation is perfectly suppressed compared with the resampling data. However, it should be noticed that the windowing operator introduces somehow a decrease in resolution, which is very sensitive to the kernel size, where resolution of the reconstructions decreases when the kernel size increases (Fig. S4**b**). Thus, in practical applications, the kernel size should be carefully selected to balance the results of robustness and resolution.


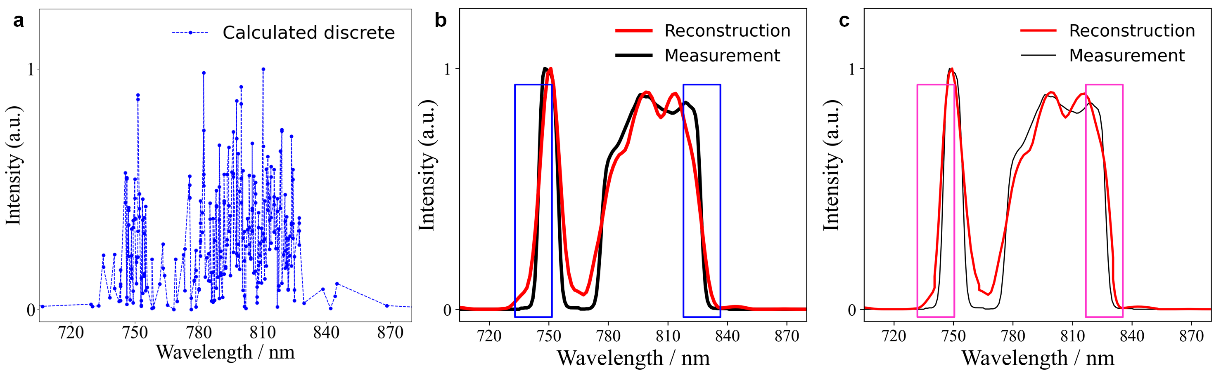


**Figure S5 a**, the calculated discrete spectrum components by the proposed diffraction-based computational spectrometer. **b**, the reconstructed spectrum mismatches with the measurement at the truncation points (blue boxes). **c**, the effect of mismatch is reduced by applying an intensive interpolation operator at the truncation points (pink boxes).

Additionally, the windowing operator may lead to matching errors in cases of transient signal, step signal, impulse response, or pulse signal applications. Fig. 5**a** gives an example of a truncated broadband spectrum reconstruction, seeing that there are mismatches at the truncation points (blue boxes). To tackle such mismatching, an intensive interpolation operator is applied to the truncation points to reduce the effects of match errors from convolution (pink boxes in Fig. S5**b**).

**Figure S6** Spectrum measurement workflow from a single-shot broadband diffraction.

**S3 Experimental set-up of the diffraction-based computational spectrometer**

**Figure S7 Experimental set up for the spectra measurements.** A supercontinuum (YSL Photonics SC-Pro-M) is used to generate an ultra-broadband spectrum from 450nm to 1100nm. A set of optical filters (Thorlabs F series) is applied to the optical path to modulate the shape of incident spectra to different spectra profiles s as mentioned in the main test. The in-lab spectrometer is very compact in size to a $\emptyset1^{"}$ lens. All the spectra are pre-measured by a Horiba iHR 550 spectrometer as the measurements in ground truth.

**S4** **Nonlinearities of detector in the diffraction-based computational spectrometer**

The proposed computational spectrometer relies on the coherent mode decomposition of broadband incoherent diffraction intensity. Since the diffraction intensity distribution exhibit an inherent characteristics of exceptionally bright central region alongside significantly dark high-order areas. This imposes critical demands on the detector for achieving high SNR during diffraction signal readout. It primarily involves three key aspects:

1. The dynamic range and spectral quantum efficiency of the detector sensor.
2. The suppression of detector noise in diffraction recording process.
3. Sensor size truncation on the active detector pixel array.

**Dynamic range.** In the context of Fraunhofer diffraction, most of the photon energy is concentrated within the central diffraction orders, while the higher-order diffracted photons are notably weaker. However, most of the redundant spectral dispersion information is mostly concentrated in the higher-order diffraction where the diffracted photons are dim. Hence, the commonly used detectors with 8-bit, 12-bit, or 16-bit analog-to-digital converter (ADC) dynamic ranges all prove inadequate in capturing the high-order diffraction signals without overexposure, as the simulations demonstrated in Fig. S8 a1~c1. To better use the detector sensor’s dynamic range, we employ a specialized filter to eliminate central fringe saturations on the detector. This leads to a significant enhanced SNR for capturing high-order diffractions, as a comparison shown in Fig. S8 a2~c2.

**
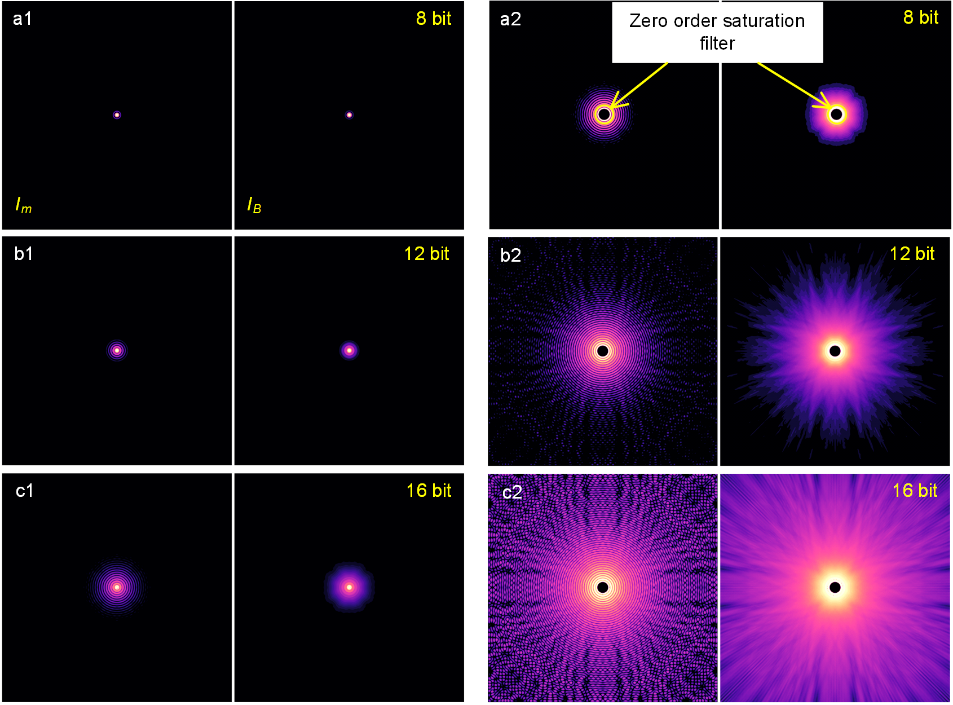
**

**Figure S8 Simulation of the detector sensor dynamic range for diffraction recording.** Rows a ~ c shows the captured monochromatic diffraction (left) and broadband diffraction (right) from the detector with varying dynamic ranges of 8 bits, 12 bits, and 16 bits, respectively. Column a1~c1 displays the under sampled diffraction data with no central stop, as a comparison in column a2~c2, a specialized filter is employed to eliminate the first several orders of overexposed diffractions.


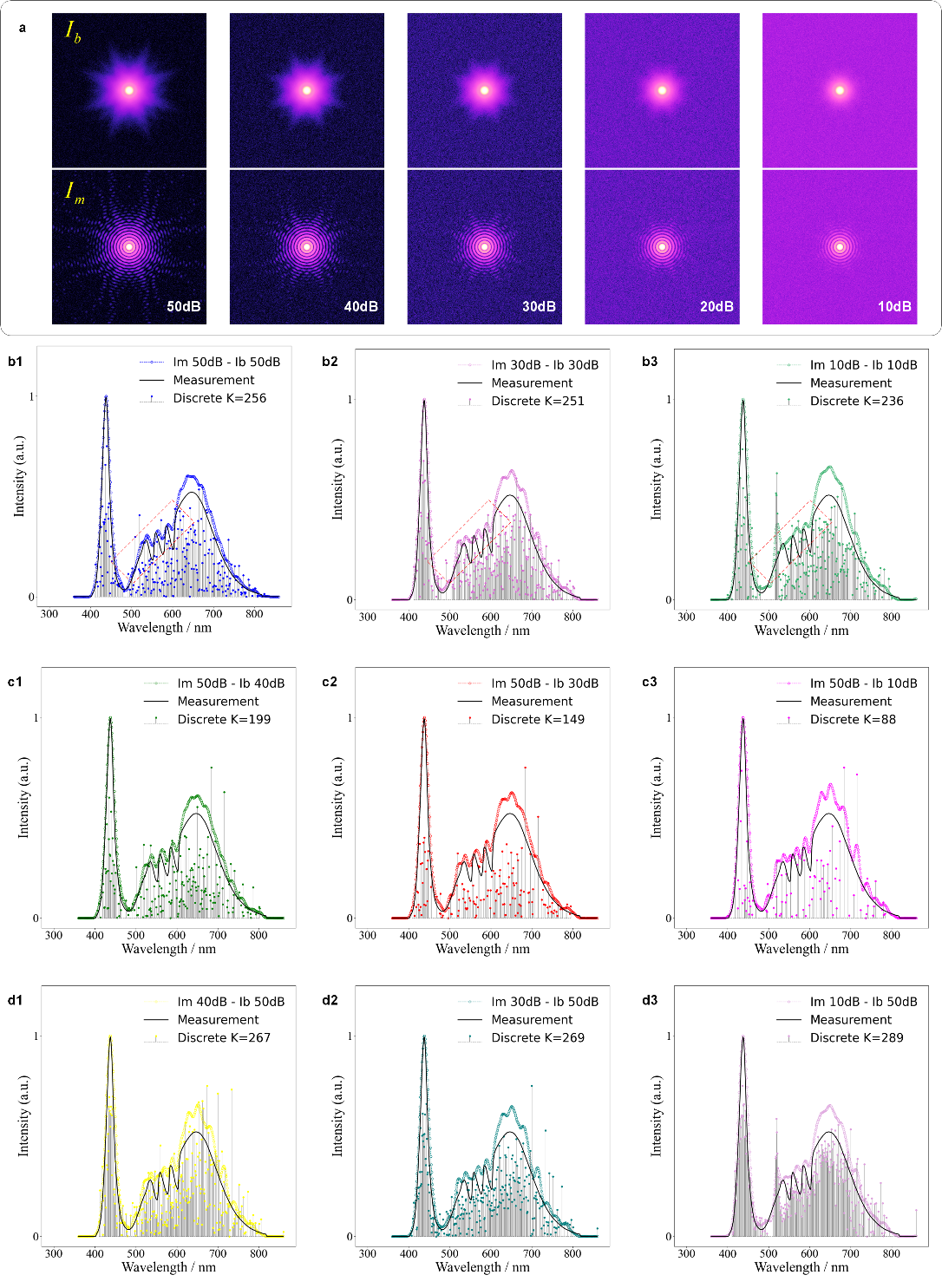


**Figure S9 Simulation for varying levels of diffraction noises in spectrum measurement. a.** varying levels of diffraction noises ranging from 50dB to 10dB (log scaled). Top row shows the corresponding broadband diffractions. Bottom row shows the monochromatic diffractions. Rows **b**~**d** plot the typical recovered spectra form the varying levels of noised diffractions in **a**, respectively. Row **b** shows the recovered spectra from the varying levels of noise in the pre-captured monochromatic diffraction and broadband diffraction ranging from 50dB to 10dB, respectively. Row **c** shows the recovered spectra from the varying levels of noise in the broadband diffraction ranging from 50dB to 10dB, respectively, while the pre-captured monochromatic diffraction is constant with 50dB noise. Row **d** shows the recovered spectra from the varying levels of noise in the pre-captured monochromatic diffraction ranging from 50dB to 10dB, respectively, while the broadband diffraction is constant with 50dB noise.

**Detector noise.** The presence of detector noise decreases the SNR in diffraction signal recording and therefore weakens the spectrum measurement accuracy. To this end, we utilized a widely-used noise model in HDR photography to analyze the primary noise sources in the acquisition of diffraction images^6,7^. The noise model includes several critical factors of camera noise, such as input scene radiant flux $Ф$, dark current $D$, sensor QE $\alpha$, exposure time$t$, readout analog voltage (with saturation), analog amplifier $g$, and ADC, which can be simply modeled as a mixture of Gaussian noise and Poisson noise.

$I\sim Poisson(t\cdot\alpha\cdot\Phi)\cdot g+Poisson(t\cdot g\cdot D)+Normal(0,\sqrt{\sigma_{read}^{2}\cdot g^{2}+\sigma_{ADC}^{2}})$ (S16)

Thus, the SNR in the detector can be qualified as the following formula:

$\mathrm{SNR}=\frac{t\cdot\alpha\cdot\Phi}{\sqrt{t\cdot\alpha\cdot\Phi+t\cdot g\cdot D+R^{2}}}$ (S17)

where R denotes the readout noise, which follows a Gaussian distribution. It's worth noting that the readout photon signal is constrained by the ADC dynamic range of the detector sensor.

Herein, we simulated detector noise in the proposed spectrometer by incorporating a combination of Gaussian and Poisson noise to the recording diffractions, with SNRs ranging from 50dB to 10dB, respectively, as demonstrated in Fig. S9 **a**. And the corresponding recovered spectra is plotted in Fig. S9 row **b**. Seeing that the proposed diffraction-based computational spectrometer reveals high robustness to noise. We observe that the reconstructed spectrum remains consistent with the ground truth, even under conditions of heavy noise, only tiny mismatches occur on steep turning points of the spectra, as indicated with the red dashed box in Fig. S9 row **b**.


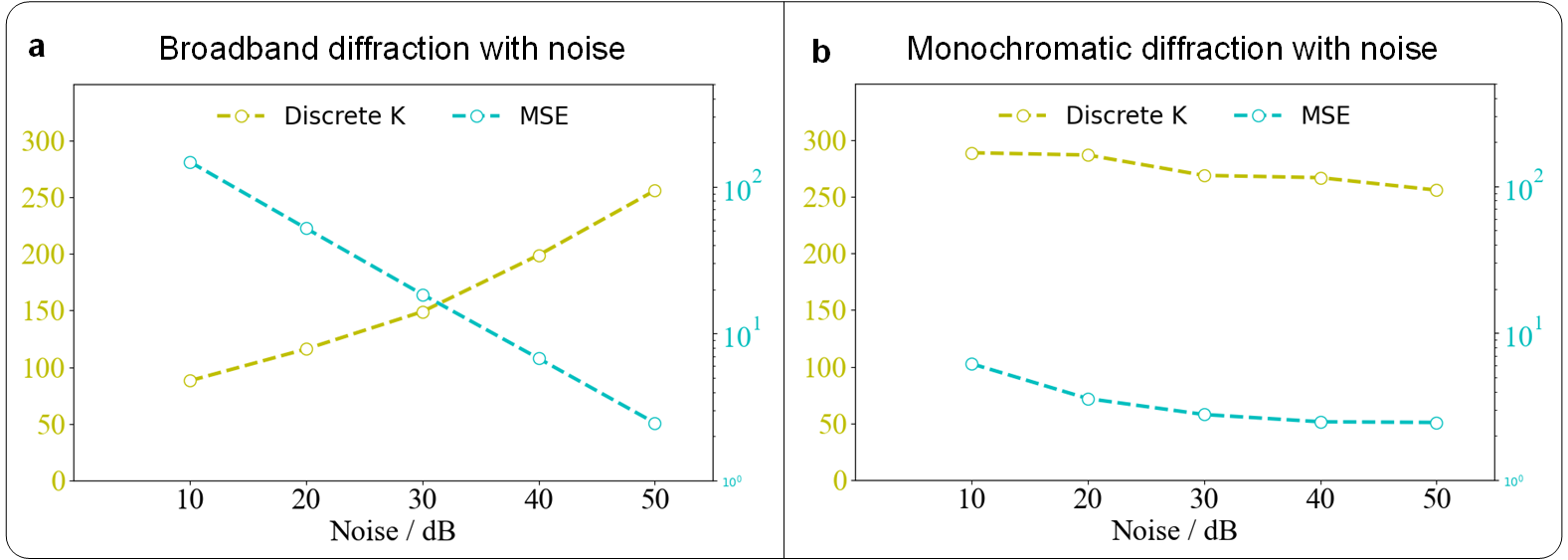


**Figure S10** Number of the calculated spectra discrete and the prediction MSE corresponding to the measurements in broadband diffraction with varying noises in **a** and monochromatic diffraction with varying noises in **b**, respectively.

Additionally, we carried out a more extensive analysis to evaluate the noise robustness in the context of recording broadband diffractions and monochromatic diffractions. Fig. S9 rows **c**, **d** presents the comparison of recovered spectra under different levels of monochromatic and broadband diffraction noises. Meanwhile, we have monitored the number of solved discrete power spectrum counts and the MSE in prediction during the spectrum calculation, as shown in Fig. S10. Seeing that the number of solved power spectrum counts increases with the noise power in broadband diffraction, whereas the MSE decreases simultaneously. In comparison, the computational spectrometer shows consistent robustness against varying noise power to the pre-captured monochromatic diffraction image. It is evident that the quality of spectrum measurement is more susceptible to noise in the presence of broadband diffraction images. This sensitivity arises from the spectrum's expansion, which results in diffraction aliasing, significantly reducing the coherence of the broadband diffraction signal and making it more vulnerable to noise. To this end, we practically employ a commonly used background noise minimization approach to suppress the detector noise in the diffraction image recording process^8^.


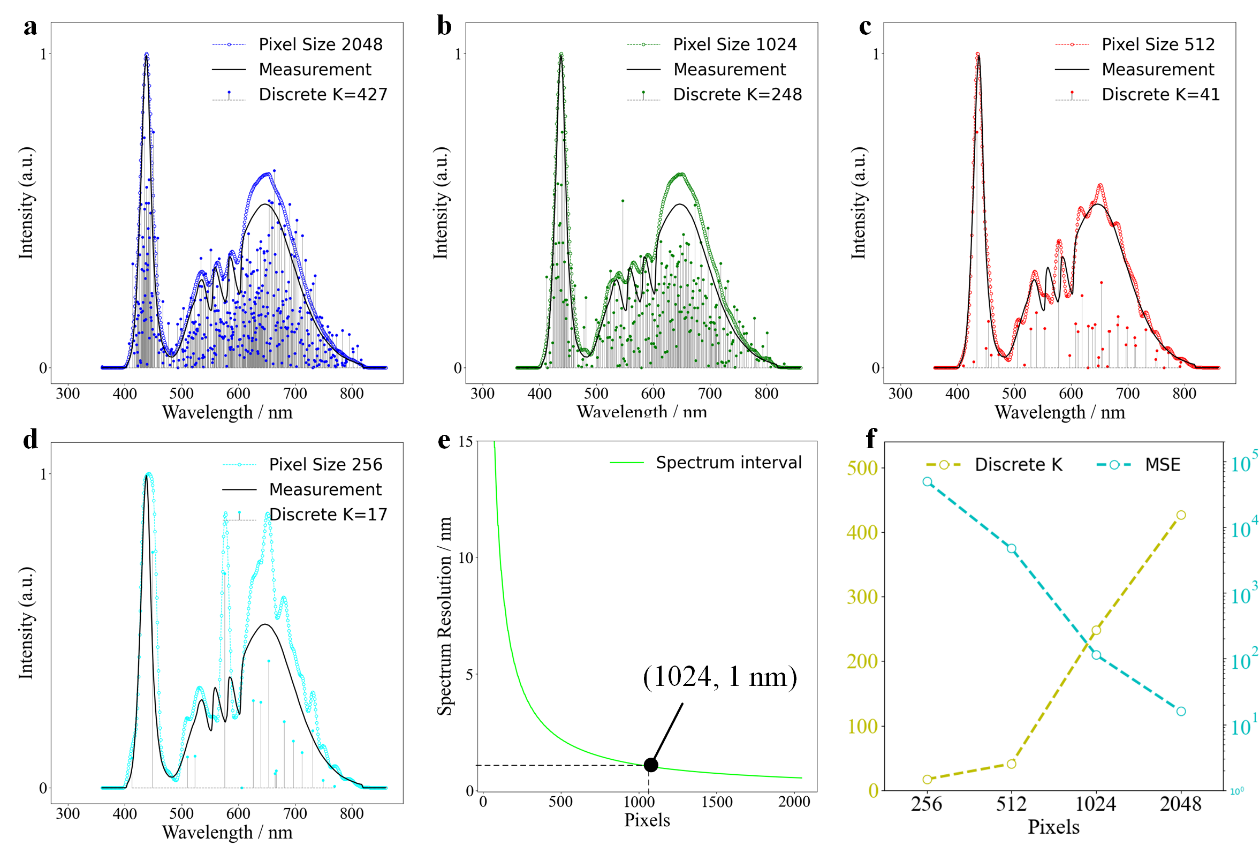


**Figure S11 Truncation effect of the diffraction pattern by the finite detector array size in spectrum measurement. a~d** showcase varying array size truncations by the finite detector in spectrum measurement. **e** plots the relationship of spectral measurement resolution with the number of sampling pixels. **f** shows the total number of the calculated spectra discrete and the prediction MSE corresponding to the measurements in broadband diffraction with varying sensor size truncations.

**Sensor size truncation.** As described in Eq. (3) in the main text, the spectral resolution of the proposed spectrometer ideally shows a reciprocal relationship with the number of sampling pixels in the active detector sensor array, primarily determined by the detector's dynamic range. In our pursuit of understanding the truncation effect caused by the finite detector array size in our spectrometer, a series of simulated analyses has been meticulously executed. As clearly depicted in Figures S11 **a**-**d**, a reduction in the effective detection target area results in fewer sampling pixels, which, in turn, leads to a marked decline in spectral measurement precision and a notable degradation in spectral peak resolution. However, the expansion of sampling pixel count results in an exponential increase in computational time for spectrum calculations. This is primarily due to a significant rise in both the number of MLE sets and the elements of discrete spectra vectors in the MLR solving model. Herein, we quantified the truncation effect of the diffraction pattern by the finite detector array size in spectrum measurement, as plotted in Fig. S11 **e**. It's evident that when the number of truncation pixels exceeds 1000, the enhancement in spectral resolution becomes less pronounced, while the computational cost significantly increases. To strike the balance between the spectrum measurement resolution and the computational cost, here we select 1024 pixels as the optimal sensor array size, providing a 1nm resolution, as demonstrated in Fig. S11 **f**.

To sum up, the camera’s nonlinearities have several key effects on the quality of diffraction recording, thereby impacting the performance of recovered spectrum, as discussed in detail in the following.

1. Quantum efficiency (QE) nonlinearity

The QE refers to the detector’s ability to efficiently convert incoming photons of broadband light into measurable electrical signals. In the proposed spectrometer, the QE of the detector is a critical factor in determining the spectrometer’s overall performance.

- Firstly, the spectral response bandwidth of the spectrometer is predominantly limited by the QE of the detector.
- Secondly, the QE nonlinearity introduces inaccuracies in the power intensity of spectral measurements. These inaccuracies can be corrected by calibrating the detector's QE.
- Thirdly, a high-QE detector has a broader dynamic range, allows to capture higher-SNR diffraction signals, and thus can improve the quality of the spectra measurements.

1. ADC nonlinearity

The ADC nonlinearity in detector refers to deviations from a perfect linear response in the process of converting analog signals into digital values. This nonlinearity introduces errors in the digital representation of the analog signal and affects accuracy of the diffraction intensity. The ADC nonlinearity also introduces inaccuracies in the measurement of spectral power intensity. Moreover, different from the QE nonlinearity, these inaccuracies resulted from the ADC nonlinearity cannot be corrected. Good news is that the performance of the CCD/CMOS sensor device has seen substantial improvement, with the ADC nonlinearity typically being reduced to less than 5% across the full QE bandwidth.

1. Saturation and clipping

The saturation and shadow clipping effects lead to a decrease in the SNR of the sampled diffraction signals. This is because most of the photon energy is concentrated within the central diffraction orders, making them prone to saturation, while the higher-order diffracted photons are much weaker and more susceptible to noise-induced blurring. As a result, these effects diminish the quality of the recovered spectra. In this work, we use a filter to remove the central fringe saturations to better utilize the ADC dynamic range of the detector.

**S5 Spectral resolution with temporal decoherence of the reference diffraction** $\boldsymbol{I}_{\boldsymbol{m}}$

As discussed in the main text, the resolution of peak separation of the bimodal spectrum degenerates from the decoherence of the quasi-monochromatic diffraction which is used to generate the PSFs over full spectrum range. This is caused by the wavelength-multiplexing of diffraction distributions of different spectral components, as described in Eq. S6. The better monochromaticity of quasi-monochromatic diffraction, the lower level of the multiplexing effect, which makes the higher resolution of spectral peak separation.

**a**

**b**

**c**

**d**

**e**

**f**

**Figure S12 Simulated resolution performance with increased temporal decoherence of pre-captured diffraction**. **a**，the simulated FWHMs of radiation for diffractions with different levels of decoherence. **b-d**, reconstructions using different pre-captured diffractions with 0.1nm, 1nm, 3nm, and 7nm FWHM, respectively. **f**, total number of the calculated spectrum discrete and the prediction MSE corresponding to the measurements in **b-d**, respectively.

To illustrate the temporal decoherence of pre-captured $I_{m}$ in resolution, we have carried out simulations for the measurement of a bimodal spectrum at a 3nm separation from the broadband diffraction corresponding to the measured spectrum, with a set of pre-captured diffractions illuminated from varying levels of FWHMs (0.1nm, 1nm, 3nm, and 7nm) as the input data, respectively, as shown in Fig. S12**a**. The reconstructions have been simulated via the proposed spectrum measurement workflow as detailed in **S2**. For the reference diffraction illuminated with a FWHM of 0.1nm, the peaks of the measured spectrum can be clearly distinguished and with high alignment with the ground truth (Fig. S12 **b**). With the FWHM increasing, the reconstructed resolution begins to break down (Fig. S12 **c**-**e**). Seeing that the peaks of the bimodal spectrum of 3nm are separated successfully for the reference diffraction illuminated with FWHMs less than 1nm, which match well with the experimental results (as demonstrated in the Figure 3**c**, and 3**d** in the main text). Meanwhile, we have monitored the number of solved discrete power spectrum counts and the MSE in prediction by solving Eq. S13, shown in Fig. S12 **e**. Seeing that the number of solved power spectrum counts decreases with the increase of FWHM, whereas the MSE increases simultaneously.

**S6 Experimental set-up of the broadband CDI**


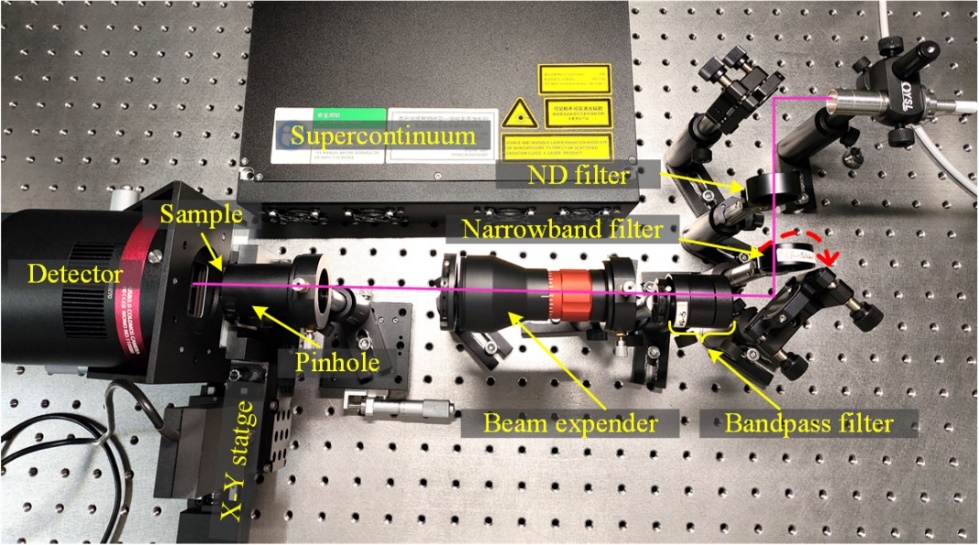


**Figure S13 Schematic of broadband CDI setup.** we use a 600 nm short pass filter to select a broadband spectrum (YSL Photonics SC-5) from the supercontinuum source. A 90° Flip narrowband filter at 532nm (Thorlabs FL532-3) is settled on the optical path to generate a quasi-monochromatic radiation. A pinhole with 100μm in diameter (Thorlabs P100K) is placed in front of the Siemens star resolution target (Thorlabs R1L1S1N) to filter the radiation to a plane wave with 100μm in diameter. A CMOS detector (QHY268M) is placed behind the sample at 30mm to record the diffractions. The sample is mounted on a X-Y stage (Thorlabs MTS25/M-Z8).

**S7 Monochromatization from a broadband diffraction**

This section describes how we monochromatize the broadband diffraction from an unknown spectrum intensity. As we have mentioned in **S1**, the wavelength-dependent scaling and weighting spectrum of copies of the pre-captured coherent diffraction pattern is perfectly suited for a matrix-vector product. As referred to Julius’s previous work on numerical monochromatization of the broadband diffraction pattern, a monochromatized diffraction pattern can be retrieved based on a regularized inversion of a matrix that depends only on the spectrum of the diffracted radiation Defining the monochromatic pattern as a vector $\mathbf{m}$, the broadband diffraction pattern as vector $\mathbf{b}$ and the scaling matrix as $\mathbf{C}$, Eq. S8 can be rewritten for simplicity as:

$\mathbf{b}=\mathbf{Cm}$ (S16)

where $\mathbf{C}$ is regarded as containing the copies of PSF in Eq. S7 times a corresponding power spectrum profile. Eq. S16 maps a point in $\mathbf{m}$ to the profiles of power spectrum in $\mathbf{b}$. For a 2D diffraction pattern, C is a 4D tensor in shape. Note that matrix $\mathbf{C}$ is fully determined by only the spectrum of illumination system and the size of detector. in 1D case of $\mathbf{C}$ can be calculated as follows:

$C_{n,j}=\sum_{\Lambda} \left[\min\left\{j,\frac{\lambda}{\lambda_{m}}n\right\}-\max\left\{j-1\text{，}\frac{\lambda}{\lambda_{m}}\left(n-1\right)\right\}\right]\frac{\omega\left(\lambda\right)\lambda_{m}}{\lambda}$ (S17)

where

$$\begin{matrix} N=\left\{n:\left(j-1\right)\min\left\{\frac{\lambda_{m}}{\lambda}\right\}<n<j\left(\max\left\{\frac{\lambda_{m}}{\lambda+\lambda_{m}}\right\}\right)\right\} \\ \Lambda=\left\{\lambda:\frac{j-1}{n}<\frac{\lambda}{\lambda_{m}}<\frac{j}{n-1}\right\} \end{matrix}$$

Since the matrix $\mathbf{C}$ is calculated from the measured spectrum $\omega\left( \lambda\right)$ combined with$\mathbf{b}$ and $\mathbf{m}$. The monochromatization of a broadband diffraction pattern can be reduced to the inversion of matrix $\mathbf{C}$ in Eq. S16. As the inversion is extremely sensitive to noise, which is generally unsolvable by ordinary noniterative methods. To mitigate this problem, a regularization method of BiCGStab scheme is performed to reconstruct the monochromatized diffraction^9,10^. BICGStab is performed with two additional constraints in this work: non-negativity of $\boldsymbol{m}_{k}$ (diffracted photon counts should not be negative) and a support constraint on the initial guess of $\boldsymbol{m}_{0}$ set to the measured broadband pattern **b**. These constraints help to prevent overfitting and further improve the regularizing power of the method.

Besides, there is a spectral deviation between the power spectrum of the original source radiation $S\left( \lambda\right)$ and the aftermost power spectrum of the diffraction pattern $\omega\left( \lambda\right)$ on the detector, since the transmittance of the sample $T\left( \lambda\right)$ or the QE of the detector $\mathrm{QE}\left( \lambda\right)$ is not constant over the source bandwidth, as

$\omega\left(\lambda\right)=S\left(\lambda\right)T\left(\lambda\right)QE\left(\lambda\right)$ (S18)

It should be mentioned that only if all these power spectrum profiles are measured precisely, Eq. S18 can be executed with satisfactory. Yet, in most practical applications, the aftermost power spectrum $\omega\left( \lambda\right)$ is generally very difficult to be measured, which brings the limitation on monochromatization of ultra-broadband diffraction applications. Our proposed spectrometer can be successfully used to tackle such instabilities, from which the aftermost power spectrum $\omega\left( \lambda\right)$ can be straightforwardly reconstructed, as detailed in S2. By performing the numerical BiCGStab algorithm to monochromatize the broadband diffraction pattern applying with the proposed spectrum reconstruction method, a monochromatized diffraction pattern can be successfully retrieved without any requirement of *prior* spectra of broadband illumination, detector QE or sample’s transmittance.

**S8 Spectral resolution with the wavelength** $\boldsymbol{\lambda}_{\boldsymbol{m}}$ **of the pre-captured coherent diffraction** $\boldsymbol{I}_{\boldsymbol{m}}$

**Figure S14 Simulated resolution performance with the wavelength** $\boldsymbol{\lambda}_{\boldsymbol{m}}$ **of the reference diffraction** $\boldsymbol{I}_{\boldsymbol{m}}$ **a**, the simulated pre-captured diffraction pattern with different radiation of spectrum from 300nm to 900nm by 100nm interval, respectively. **b**, the broadband spectrum in simulation and the quasi-monochromatic spectrums corresponding to Fig. S14 **a**, orange dotted line plots the mass of the center of the broadband spectrum. **c-i**, reconstructions using different pre-captured diffractions in **Fig.** S14 **a** at wavelengths in Fig. S14 **b**, respectively. **j**, total number of the calculated spectrum discrete and the prediction MSE corresponding to the measurements in Fig. S14 **c -** Fig. S14 **i**, respectively.

Relating to our computational strategy, as detailed in section S2, to reconstruct the full spectrum from the corresponding single-shot broadband diffraction $I_{B}$, we used a PSF mapping scheme to generate $n$ slices of wavelength-dependent quasi-monochromatic diffraction components by a uniform spectrum interval $\delta\lambda$ over full spectrum range from only one shot of pre-captured coherent diffraction $I_{m}$ at a wavelength $\lambda_{m}$. As described in Eq. S7, the PSF mapping scheme is performed by scaling the reference diffraction $I_{m}$ with a wavelength-dependent factor ${\lambda_{i}}/{\lambda_{m}}$. Thus, an interpolation approach is required to be implemented on the PSFs to match with the broadband diffraction $I_{B}$ with the same pixel size, which may introduce interpolation errors to the reconstructions. It is obvious that the level of interpolation errors is associated with the wavelength-dependent scaling factors, the values of ${\lambda_{i}}/{\lambda_{m}}$ closer to 1, the smaller the interpolation errors. Thus, the choice of wavelength $\lambda_{m}$ should better be around of the mass of the center of the broadband spectrum to suppress the interpolation errors in PSF mapping.

To illustrate the interpolation error of PSF mapping in reconstructed resolution, we have carried out simulations for the measurement of an ultra-broadband spectrum from 350nm to 850nm (black line in Fig. S14 **b**) with a series of pre-captured quasi-monochromatic diffractions at wavelengths from 300 nm to 900 nm by 100nm interval, respectively, as shown in Fig. S12 **a**, and the corresponding spectrum distributes in Fig. S14 **b**. Additionally, we calculated the weighted spectrum center of the broadband spectrum at 580 nm, as plotted with orange dotted line in Fig. S14 **b**. The calculated discrete results are plotted in Fig. S14 **c**- Fig. S14 **i**, respectively. Simulations shows that the results calculated from the pre-captured diffraction at a 600 nm wavelength, which is closest to 580nm, have the best resolution in measurement. With the wavelength of the pre-captured diffraction being father from the weighted spectrum center of the broadband spectrum, the reconstructed resolution begins to break down. Meanwhile, we have monitored the number of solved discrete power spectrum counts and the MSE in prediction by solving Eq. S13, shown in Fig. S14 **j**. Seeing that the number of solved power spectrum counts increases with the wavelength of the pre-captured diffraction close to the weighted spectrum center of the broadband spectrum, whereas the MSE decreases simultaneously.

**References**

1 Ioseph W. Goodman. *Introduction to Fourier Optics*. 4th ed. Roberts and Company Publishers, 2005.

2 Wang C, Xu Z, Liu H, Wang Y, Wang J, Tai R. Background noise removal in x-ray ptychography. *Appl Opt* 2017; **56**: 2099.

3 Gonzalez RC, Woods RE, Eddins SL. *Digital Image Processing Using MATLAB*. 3rd editio. Gatesmark Publishing, 2020.

4 Xu Y, Pei Y, Dong F. An adaptive Tikhonov regularization parameter choice method for electrical resistance tomography. *Flow Meas Instrum* 2016; **50**: 1–12.

5 Golub GH, Heath M, Wahba G. Generalized cross-validation as a method for choosing a good ridge parameter. *Technometrics* 1979; **21**: 215–223.

6 Hasinoff SW, Durand F, Freeman WT. Noise-optimal capture for high dynamic range photography. In: *2010 IEEE Computer Society Conference on Computer Vision and Pattern Recognition*. IEEE, 2010, pp 553–560.

7 Healey GE, Kondepudy R. Radiometric CCD Camera Calibration and Noise Estimation. *IEEE Trans PAlTERN Anal Mach Intell* 1994; **16**.

8 Wang C, Xu Z, Liu H, Wang Y, Wang J, Tai R. Background noise removal in x-ray ptychography. *Appl Opt* 2017; **56**: 2099–2111.

9 van der Vorst HA. Bi-CGSTAB: A Fast and Smoothly Converging Variant of Bi-CG for the Solution of Nonsymmetric Linear Systems. *SIAM J Sci Stat Comput* 1992; **13**: 631–644.

10 Tibshirani R. Regression Shrinkage and Selection via the Lasso. *J R Stat Soc Ser B* 1996; **58**: 267–288.
